# Contrastive multi-view representation learning for multi-camera plant phenotyping: A cotton field study

**DOI:** 10.1016/j.plaphe.2026.100193

**Published:** 2026-03-06

**Authors:** Daniel Petti, Changying Li, Ninghao Liu

**Affiliations:** a Bio-Sensing, Automation, and Intelligence Laboratory, Department of Agricultural and Biological Engineering, Institute of Food and Agricultural Sciences, University of Florida, 1741 Museum Rd, Gainesville, 32603, Florida, USA; b School of Computing, University of Georgia, 200 D. W. Brooks Drive, Georgia, Athens, 30602, USA

**Keywords:** High-throughput phenotyping, Machine vision, Self-supervised learning, Contrastive learning, Cotton yield estimation, UGV

## Abstract

Attempts to deploy computer vision in agricultural tasks often suffer from a shortage of annotated data. One strategy to alleviate the impact of limited data is Self-Supervised Learning (SSL), which involves pre-training a model on a pretext task that utilizes automatically generated annotations. The primary objective of this study is to leverage a multi-camera view dataset of cotton boll images for contrastive learning in order to enable phenotyping tasks with minimal data annotation. This dataset was collected in the field using six camera views. The efficacy of two contrastive learning frameworks (SimCLR and MoCo) in producing representations when positive examples originate from different cameras was investigated, and a comprehensive study of how the camera positions affect performance was conducted. After self-supervised pre-training, linear evaluation and semi-supervised learning experiments were performed on boll detection and plot status downstream tasks. In general, using multiple camera views with SimCLR and MoCo improves cotton boll detection mean average precision by **14%** compared to vanilla SimCLR and MoCo. Through careful investigation using synthetic data, it was determined that relative camera poses with an intermediate amount of overlap seem more likely to perform well. Neither MoCo nor SimCLR was consistently superior to the other in this context. The representations embed meaningful features about the cotton plants, such as overall boll density, but also less meaningful ones, such as lighting variations. This technique could potentially accelerate the development of phenotyping algorithms based on data collected from field robots.

## Highlights

• A contrastive learning method based on comparing multi-camera views was developed.
• The method was tested with images of cotton bolls from a ground robot.
• The method outperformed baseline contrastive learning approaches.

## Graphical abstract

**Figure:**
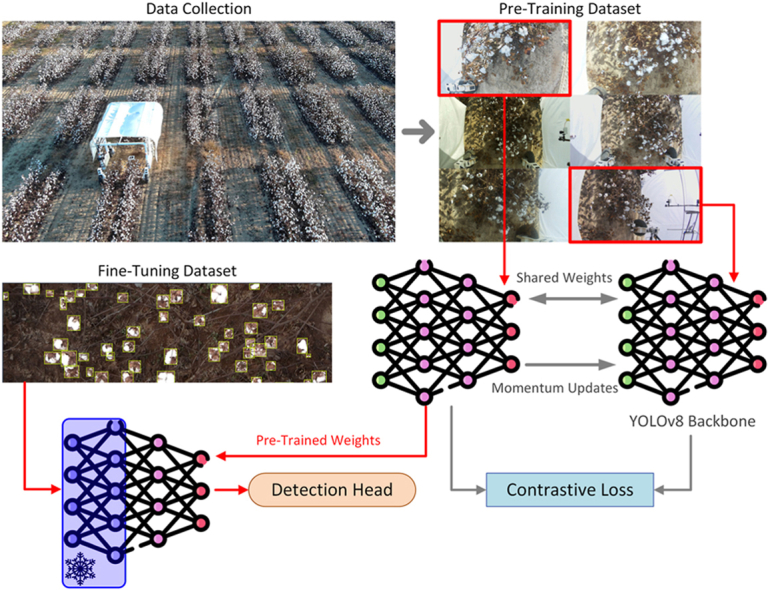

## 1 Introduction

High-Throughput Phenotyping (HTP) from digital images is an area of active research that seeks to accelerate the laborious manual process of plant phenotyping by reducing labor requirements. Due to the difficulty of extracting useful trait information from raw images, many modern approaches leverage deep learning techniques which have been proven to solve complex vision problems in other domains. For instance, although automatically counting plant organs from images has been an active area of research for several decades [1], modern deep-learning based methods have proven quite effective on both proximal [[2], [3], [4], [5], [6], [7], [8], [9], [10], [11], [12], [13]] and remote sensing data [[14], [15], [16], [17], [18]]. In order to be effective, however, traditional deep learning requires significant amounts of annotated data which can be tedious and time-consuming to collect. To mitigate this, some methods use techniques such as active learning [17,19], learning from simulated data [9,20], and various types of weakly-supervised learning [2,14,17,[21], [22], [23]]. Though these approaches successfully reduce labeling requirements, they also tend to sacrifice some accuracy and to be highly tailored to specific crops, limiting their overall adoption. For example, although several counting methods that are specific to cotton blooms have been proposed recently [5,11,17,18], most of them are based on large hand-annotated datasets.

Self-Supervised Learning (SSL) is a family of approaches for reducing the reliance of deep learning on human-annotated data. SSL methods define a proxy task, such as random rotation [24], colorization [25,26], ranking the number of objects in image crops [21,27], image in-painting [28,29], or random augmentations [30], which is employed to automatically generate annotations from unlabeled data. In theory, excelling at the proxy task forces the model to learn semantically meaningful features which can then be used as a basis for transfer learning on a much smaller amount of labeled data. In practice, on large datasets such as ImageNet [31], self-supervised pre-training has historically been surpassed by fully-supervised pre-training [30,32]. Therefore, despite its long history [33], for decades, SSL did not see much use in state-of-the-art computer vision models. In the past few years, this changed radically with the introduction of vision foundation models [34,35] which are trained on Internet-scale datasets for which human annotations are entirely impractical [36]. In fact, SSL has shown particular promise with datasets that are challenging to annotate, such as those that are highly multi-modal [[37], [38], [39]] or require domain-specific knowledge [40,41]. Although recent SSL approaches have challenged its dominance [28,29], contrastive learning remains one of the most common techniques in this field [42]. Indeed, theoretical analysis [43] suggests that contrastive learning is sufficient to learn useful representations. The archetypal contrastive learning approach for images remains SimCLR [30], likely due to its simplicity and ease of implementation. Recently, more advanced contrastive frameworks have been proposed [32,44,45] which can offer improved performance in some situations. Additionally, performance improvements can be realized by combining contrastive learning and non-contrastive SSL approaches such as Masked Auto-Encoders [28,29,46] into a unified framework [34,47].

A general idea in contrastive representation learning is to contrast multiple “views” of the same scene [48]. Views can constitute different components of a single image (e.g. L and ab), or different modalities (e.g. RGB + Depth, or RGB + text descriptions). For instance, one study leveraged a dataset with rich contextual information to select positive examples that are similar in both location and time [49]. SimCLR [30] uses random augmentations, such as cropping and color jitter, to produce two different augmented versions. These become a positive pair for the purposes of contrastive learning, and the model learns to associate these augmented images. However, choosing the proper augmentations when generating a positive pair is a common challenge in contrastive learning. Experimental evidence suggests that the chosen augmentations can have an outsized effect on performance [30,50]. The general understanding is that the views making up a positive pair must be sufficiently similar (in more technical terms, have a similar amount of mutual information), but not *too* similar [51]. In other words, there is an optimal threshold for the similarity between the views used as positive pairs in contrastive learning, and selecting the proper amount (and type) of data augmentation is a non-trivial issue.

Some recent digital agriculture studies have used modern SSL techniques to alleviate limited data availability. This can be particularly useful for remote sensing, where SSL has been applied to yield prediction [52], biomass estimation [53], crop field detection [54], and fine-grained semantic segmentation [55]. Of these approaches, three used contrastive learning [52,54,55], and one used a custom, non-contrastive method [53]. SSL is often suitable for these applications because large amounts of unannotated data are available, despite annotated datasets being relatively limited, and repeat visits by the satellite allow for the leveraging of temporal correlations [55]. SimCLR [30] has been used as a pre-training step for cherry maturity detection [56] and cucumber disease recognition [57]. The latter is a hybrid approach that also fine-tunes CLIP [58] for image-text alignment. Similarly, CLIP and DINO [59] form the basis of AgriCLIP [60] which is a vision-language foundation model for agricultural tasks. Despite these advantages, SSL has seen scant applications for proximal phenotyping of row crows, despite the existence of the same data annotation limitations. Generic SSL approaches may struggle in this scenario due occlusions by the canopy, intense lighting variation, and the fact that all target plants have similar appearances leading to a relatively weak signal for contrastive learning. Furthermore, it is relatively easy to add multiple synchronized cameras to field data collection platforms, which is a generally accepted method of mitigating occlusions. It follows that existing agricultural SSL methods, which generally do not leverage multi-camera correlations, are possibly leaving performance on the table.

In addition to SSL, synthetic data generation can also be used to enhance the performance of data-hungry deep learning models on agricultural tasks [[61], [62], [63]]. Synthetic data are often generated using traditional rendering techniques, but can be made more realistic through the use of deep learning [64,65]. The use of synthetic data for self-supervised learning, however, is less well-explored. Both SSL and synthetic data are methods of reducing the need for annotated data, and in most situations, one will suffice. Using both is only really discussed in contexts where obtaining even a sufficient volume of *unannotated* data is challenging, such as large language model development. This is why the primary use of synthetic data in this study is as a method of controlling extraneous variables in particular experiments as opposed to a complete replacement for real data.

**Fig. 1:**
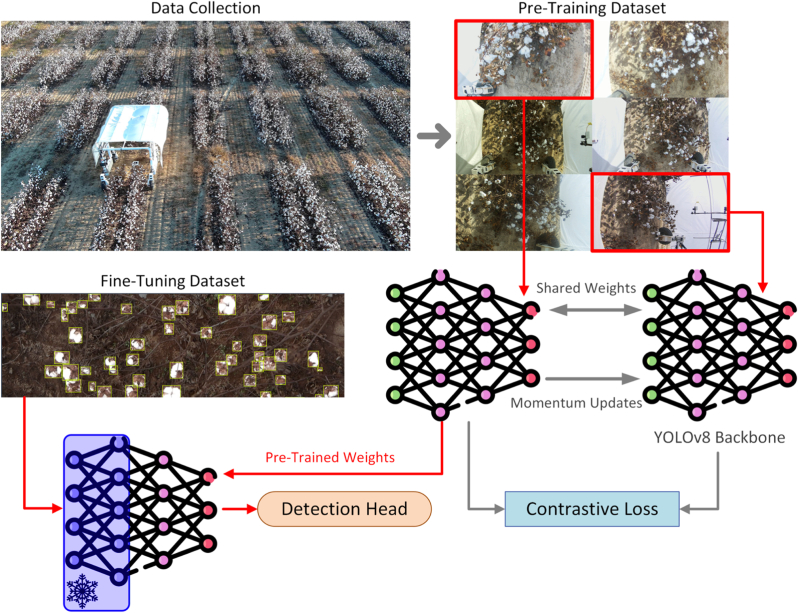
The proposed method leverages a dataset of cotton boll images collected using a field robot equipped with six cameras, which are then used as input for a multi-camera contrastive learning technique using both MoCo [45] and SimCLR [30]. To evaluate the efficacy of this procedure in a “real-world” scenario, a YOLOv8 object detector is pre-trained using contrastive learning, and then partially fine-tuned on a small dataset with hand-annotated cotton bolls.

The overall goal of this study is to investigate the feasibility of multi-camera view self-supervised learning for several plant-phenotyping-related downstream tasks and datasets. This paradigm takes advantage of the fact that phenotyping robots often employ multiple cameras to capture the entire canopy of the plants with minimal occlusions [66,67]. While a few studies propose using data from multiple cameras for contrastive learning [48,49,[68], [69], [70]], none have examined the applicability of such approaches to agricultural tasks. This is despite the fact that multiple-camera systems are prevalent on agricultural robotic platforms, in which they are often used to alleviate occlusions of plant organs [5,[71], [72], [73], [74]] or track objects over a long distance [75]. In fact, self-supervised learning as a whole remains underexamined in the agricultural domain, despite a general acknowledgment that traditional fully supervised learning is often not optimal [14,17,19,21]. Specifically, the proposed method uses views of the same scene captured at the same time but from different cameras. This is possible because the dataset used for SSL pre-training was collected with a robotic platform incorporating six overlapping cameras (Fig. 1). In theory, this will yield better performance than generating “artificial” positive pairs through data augmentation, as is traditional for contrastive learning approaches [30]. In order to evaluate the performance of this technique, a standard object detector (YOLOv8) is pre-trained using contrastive learning and then partially fine-tuned on a small hand-annotated dataset. An additional goal is to systematically assess the effects of camera placement on model performance, for which a novel synthetic dataset—modeled after the real data—was employed in order to limit the number of confounding variables.

The specific objectives of this study were to:

1. Explore two contrastive learning approaches with boll detection and plot status prediction as representative downstream tasks;
2. Rigorously investigate the optimal camera overlap in multi-view contrastive learning using synthetic data.
3. Verify the model's ability to learn semantically meaningful representations in the latent space.

## 2 Methods

### 2.1 Dataset

#### 2.1.1 Real data

To fully leverage the benefits of contrastive learning techniques, we construct a large dataset of unannotated videos of cotton plants. These videos were collected on October 25th, 2024, using the MARS-X robotic platform [67]. The robot was deployed in a cotton field at Gibbs Farm, in Tifton, Georgia, USA, after the plants had been defoliated. This field used 6-foot row spacing, and the robot was used to scan 114 plots of varying genotypes. The robot operated autonomously during this time, following a pre-programmed path through the field. This path did not include any backtracking, such that the robot scanned every plot in the field exactly one time. This ensures that every frame in the dataset was taken from a unique position, which will later facilitate the process of contrastive learning.

**Fig. 2:**
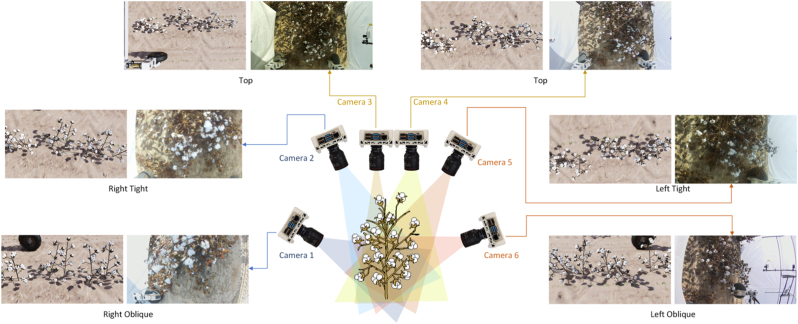
Video data is captured from six cameras at six different angles. Example images are shown from each camera view for both the real (right) and synthetic (left) datasets.

Videos from MARS were captured using an array of six Raspberry Pi HQ cameras (Raspberry Pi Ltd, Cambridge, U.K.) at a resolution of 1080p. The cameras were arranged on all sides of the plants, with two each on the right and left sides, as well as two on the top. The two cameras on the top captured mostly overlapping field of views, and were used in this study to disentangle the effect of using images captured from different view-points from that of merely using images from different cameras. The cameras on each side consisted of a “tight” view, which was only slightly off nadir, and an “oblique” view, which captured a much more side-on image (Fig. 2). For the purposes of this study, cameras were numbered as shown in Fig. 2. The clocks on the six cameras were synchronized using the Precision Time Protocol (PTP), ensuring precise timestamps for every captured frame. When constructing the dataset, the timestamps for each frame used in a positive pair are allowed to differ by a maximum of 50 ms.

The complete dataset contains 343,678 individual frames encompassing all six views. Although a canopy was used during data collection to maintain diffuse lighting, there are still significant lighting variations in the data due to the sun being low in the sky during data collection. The original videos were captured at a frame rate of 30 FPS. The dataset is organized into groups of synchronized frames extracted from the raw videos at each time step.

One known feature of contrastive learning is that the dataset's construction can significantly impact performance [76], therefore, attention to detail is necessary to ensure the quality of the data. During data collection, the ground vehicle moved forward at a constant speed, so all videos were expected to exhibit fairly uniform motion. However, in certain conditions such as the beginning and end of a video, or when a hardware fault was detected, the vehicle may have remained stationary while the cameras were recording. To eliminate the resulting frames, a simple motion filter was applied: consecutive frames were converted to grayscale and slightly blurred, and then the average absolute difference was calculated between all the corresponding pixels. If this number did not exceed a certain threshold, the later frame was discarded from the dataset.

For contrastive learning from images, the entire dataset is treated as a collection of frames. In the simplest case, a frame is then randomly selected from the dataset and random data augmentation is applied in order to generate a positive pair. After self-supervised pre-training, three additional downstream datasets are used for evaluation (Table 1, rows 2 and 3). The *detection eval* dataset comprises 290 images collected with the MARS-X robot during 2022. 116 images are used for fine-tuning, and the rest are used for validation. These images were taken from a nadir view with a DSLR camera and come from a field at the Iron Horse Farm in Watkinsville, GA. Bounding-box annotations are provided for all images in this dataset. Small datasets are deliberately used in order to test the ability of the SSL technique to reduce annotation requirements.

**Table 1:** Overview of the datasets used in this study. The first two datasets are used for self-supervised pre-training, and the others are used only for supervised fine-tuning and evaluation.

Dataset	Annotations	Year	Training Images	Testing Images	Use Case
MARS-X Data	None	2024	343,678	N/A	SSL Pre-training
Synthetic Data	None	N/A	11,340	N/A	SSL Pre-training
Boll Detection	Box	2022	116	174	Downstream eval
Plot Status (Defoliated)	Class	2022	50	20	Downstream eval
Plot Status (Foliated)	Class	2021-22	50	20	Downstream eval

**Fig. 3:**
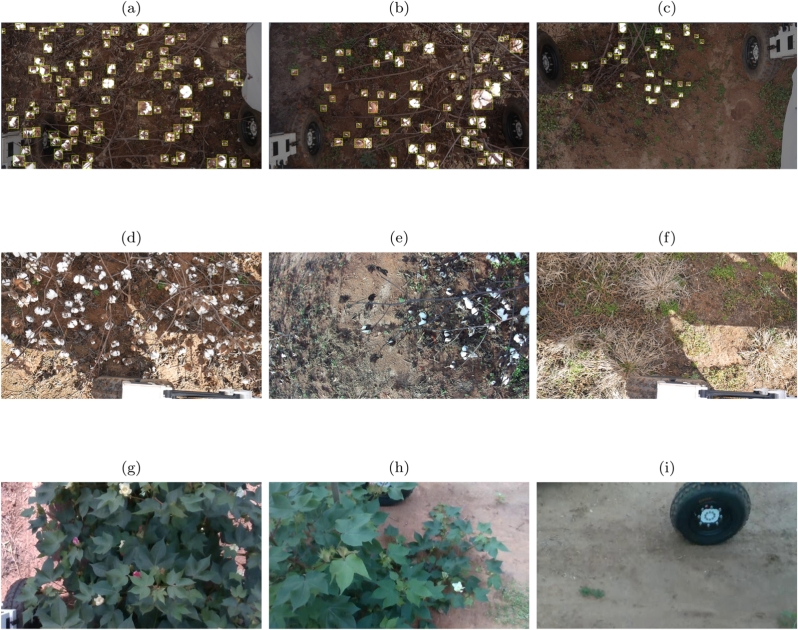
Example images from the validation datasets. The top row shows examples from the boll detection dataset. The middle row show examples from the *plot status* dataset. The bottom row shows *plot status* data from before defoliation. From left to right, the images in the bottom two rows fall into the *within plot*, *between plots*, and *headland* classes.

This study also introduces the *plot status* dataset, which provides a simpler way to evaluate the quality of the representations being learned by the method. This is a classification dataset comprising a selection of images randomly sampled from 2022 data, in which each image is classified into one of three classes: outside row, within plot, or between plots (Fig. 3, top row). Each of the three classes is defined by what is visible in the camera view. A particular image falls into the “within plot” category if the canopy is visible and the edges of the plot are not visible (Fig. 3d and g). If the edges of the plot *are* visible, then it falls into the “between plots” category (Fig. 3e and h). Finally, if none of the canopy is visible at all, it should be categorized as “headland” (Fig. 3f and i). As data is collected by moving down a row in the field, it is often useful to know where the boundaries of each plot are, such that per-plot analyses can be performed on the data. Traditionally, such per-plot data extraction is a tedious manual process, and automation with computer vision would be desirable. Fine-tuning is **not** used to evaluate on this dataset; instead, the linear evaluation protocol [30] is used, in which a linear classifier is trained using the learned representations from the pre-trained model as input.

A version of the plot status dataset is created which contains images of the plants taken prior to defoliation, during the flowering period (Fig. 3, bottom row). This dataset is used to test the generalization ability of the models, specifically, how well a model trained on defoliated plants performs on plants with foliage. The dataset contains images from three growing seasons, from 2020 through 2022, and is therefore very diverse.

#### 2.1.2 Synthetic data

In order to more rigorously analyze the effect of camera positioning on SSL performance, a dataset of synthetic cotton boll imagery was created. This was necessary because it is difficult to fully control all of the confounding variables—such as lighting, wind, weed presence, the presence of lodged plants, etc.—when collecting data with real cameras. The dataset was generated using Nvidia Isaac Sim, and was based on a model of the cotton field with 25 plots. Each plot is populated with procedurally-generated cotton plants, created using a script in Blender. An accurate CAD model of the MARS-X robot was used, and was configured to drive through the simulated field while collecting data from 6 simulated cameras. An attempt was made to position these cameras in a manner that mirrored the camera configuration used in the real dataset (Fig. 2). In total, 346,770 images were generated in this fashion, and were then sub-sampled to 11,340 in order to ensure that all the frames used in the dataset were significantly different from each-other. The soil was simulated based on a texture and surface model computed from UAV imagery of an actual field. Overall, the synthetic images were significantly more homogeneous than the real ones, allowing for the elimination of extraneous variables. Examples of the synthetic images are shown in Fig. 2.

### 2.2 Multi-view contrastive learning

**Fig. 4:**
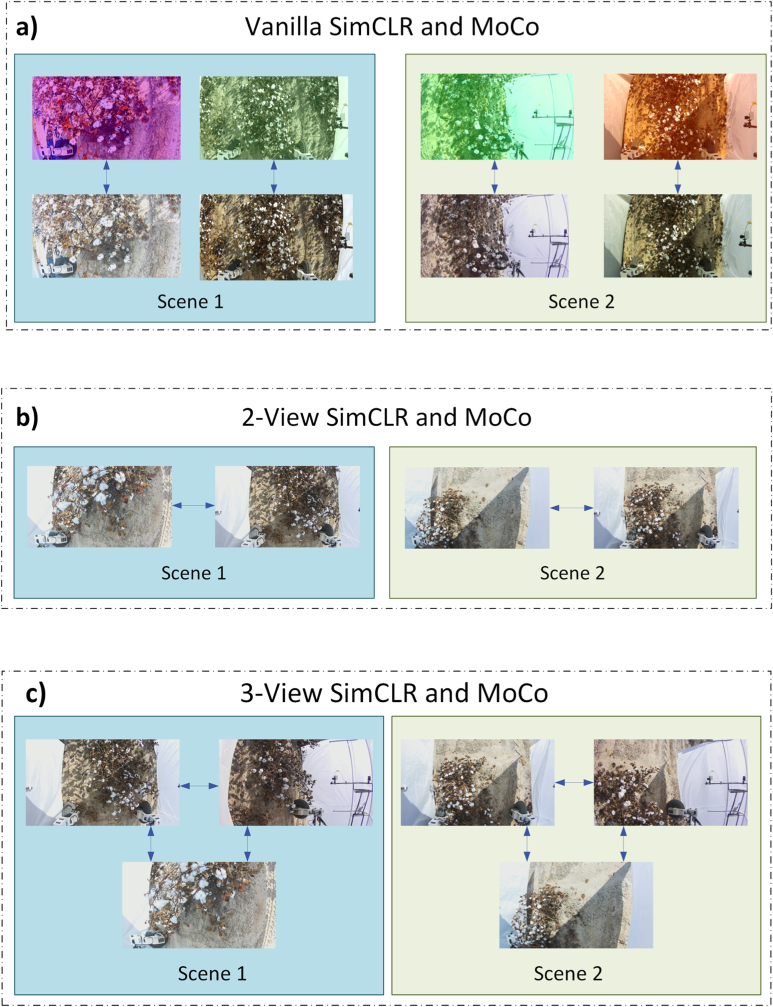
The composition of a batch for variations of SimCLR and MoCo. Arrows denote positive pairs, while all other pairings within the batch are negative. Two example scenes are shown, captured with the same camera array at two different timesteps. Vanilla SimCLR and MoCo (a) create positive pairs by augmenting (cropping) single images. Instead of augmentations, we can use two camera views of the same scene (b). If we have more than two views (c), each image is part of multiple positive pairs.

For contrastive learning, the terminology of Chen et al. [30] is adopted, where *positive pairs* refer to semantically similar pairs of views which should ideally have the same representations, and *negative pairs* refer to semantically different views which should ideally have different representations. The goal of contrastive learning is to maximize the similarity between the representations of positive pairs while simultaneously minimizing the similarity between negative pairs. In the original SimCLR and MoCo frameworks, positive pairs are generated by applying random augmentations to a single image, and negative pairs are generated by sampling two random images from the dataset (Fig. 4a). By contrast, in the proposed multi-view contrastive learning framework, positive pairs constitute images taken at the same time by two cameras with overlapping fields of view, and negative pairs are randomly sampled from completely different timesteps (Fig. 4b and c).

Nominally, the construction of the proposed multi-camera dataset presents a slight problem: the losses used in contrastive learning techniques are usually designed to compare only two views, but up to six views are potentially available. A naive, though perhaps sub-optimal, way to address this is simply to randomly select two cameras from the pool of six for every training example (Fig. 4a). In practice, however, care should taken to ensure that *all* possible combinations of different camera views are treated as positive pairs. For instance, if three cameras are considered, each image from camera one will be part of one positive pair with a corresponding image from camera two, and another with an image from camera three (Fig. 4c). To put it succinctly, with more than two views, each example in the mini-batch can be part of multiple positive pairs. This violates the general assumption with SimCLR that each batch of size 2*N* contains only *N* positive pairs, one for each augmented example in the batch.

For vanilla SimCLR, the *NtXent* loss is applied to the two representation vectors produced from two views of the same scene (*z*_*i*_, *z*_*j*_), selected from a batch containing 2*N* images (Eq. (1)). As in Chen et al. [30], cosine similarity is chosen for the *sim*() function, and a value of 0.1 is selected the temperature parameter *τ*.

(1) $$l_{i,j}=-log\frac{exp\left(sim\left(z_{i},z_{j}\right)/\tau \right)}{\sum_{k=1}^{2N}1_{\left[k\neq i\right]}exp\left(sim\left(z_{i},z_{k}\right)/\tau \right)}$$

For the proposed multi-camera SSL technique, this is generalized to *V* views, such that a total of *V* × *N* images are present in the batch and NV2 positive pairs are observed. Like vanilla SimCLR, negative examples for a particular image are generated from every other image in the batch, excluding other cameras at the same timestep (Fig. 4c). Though Eq. (1) still applies, the main difference in the loss calculation is that more positive examples are present in each batch, owing to the V2 positive pairs generated by each group of *V* camera views from a given timestep.

One advantage of this formulation is that it allows for a relatively constant number of negative examples, even as the number of views (*V*) increases and *N* necessarily decreases due to memory constraints. Previously, Chen et al. [30] indicated that the number of negative examples present in each batch impacts performance. However, it should be noted that these negative examples might still be of lower quality with more views, as different camera views from the same timestep are likely to exhibit high semantic similarity.

Ultimately, MoCo [45] is considerably less complicated when it comes to using more than two views. This is because representations for negative examples are sampled by MoCo from a queue, decoupling them from the batch size and allowing many negative examples to be used, even with small batches. Therefore, there is less need for concern regarding the number of positive and negative pairs within one batch.

### 2.3 Self-supervised pre-training with MoCO and SimCLR

Depending on the overlap between camera fields-of-view, the multi-camera SSL task can be quite difficult. In theory, it should cause the contrastive learning method to prioritize the learning of features that are invariant across camera angles. It could, however, also prevent it from learning anything at all.

For both MoCo and SimCLR, multi-camera approaches are compared against several strong baselines. In particular, versions of the same model are trained on the same cotton boll dataset but with the vanilla data augmentation procedure used to generate positive pairs. A version of the YOLOv8-L model pretrained on COCO is also included, to allow comparison against-supervised pre-training.

Initial experiments are performed with MoCo [45] in order to establish the fundamental nature of multi-camera contrastive learning. First, an experiment is performed to test whether it is advantageous to still apply additional data augmentation, even when multi-camera data is available. In this experiment, two different versions of the model are pre-trained on the cotton boll dataset with MoCo, both of which use all six available camera views. One model is trained with additional data augmentation, and the other is trained without.

Collecting data from six cameras in the field is cumbersome. Therefore, experiments were performed to determine whether a lesser number of cameras with carefully chosen fields of view will suffice. For these experiments, three additional versions of the model are pre-trained with MoCo, each of which uses three cameras instead of all six. Specifically, both combinations of the top and two side views (oblique and tight), as well as all of the cameras on one side (top, right tight, and right oblique) are tested.

It is hypothesized that SimCLR and MoCo will be significantly affected by the number of views used for training. To test this, models are pre-trained with every possible number of views, from 1 to 6. The selection of views at each stage is informed by the results of the initial MoCo experiments. In other words, the best-performing 2-view combination, as well as the best-performing 3-view combination, are tested. The remaining views are added one-by-one for the 4, 5, and 6-view experiments.

For all experiments that use data augmentation, including the baselines, it is performed by randomly selecting crops from each input frame and resizing them to 410x410 pixels. The *RandAugment* approach [77] is then applied using two random augmentations with a magnitude of 2. Otherwise, the data augmentation is kept as close as possible what was used in the original studies.

### 2.4 Quantifying camera overlap with synthetic data

**Fig. 5:**
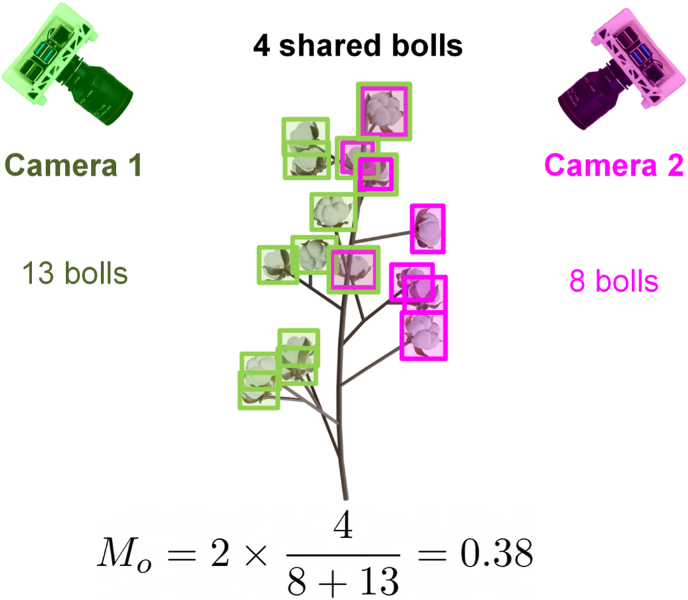
Illustration of how *M*_*o*_ is calculated on the synthetic data. Two cameras both view the same plants, and see different, but overlapping sets of bolls.

Evaluating the effects of camera placement on SSL performance requires a quantitative method of comparing camera positions. Merely measuring the Euclidean distance between cameras is insufficient, as this has almost zero correlation to SSL performance. Instead, a metric based on boll visibility is proposed. Specifically, this metric is calculated on corresponding images from two cameras, and is computed as:

(2) $${M_{o}}^{cam_{1},cam_{2}}=2\times \frac{\left|B_{cam_{1}}\cap B_{cam_{2}}\right|}{\left|B_{cam_{1}}|+|B_{cam_{2}}\right|}$$

where $B_{cam_{1}}$ is the set of unique bolls visible in the first camera view and $B_{cam_{2}}$ is the set of unique bolls visible in the second camera view. The multiplication by 2 is to put the metric in the range (0, 1), where a value of 0 indicates that no bolls are visible in both cameras, and a value of 1 indicates that every boll is visible in both cameras. Fig. 5 shows a visual representation of this metric.

A primary advantage of the synthetic dataset is that this metric is easy to compute. Isaac Sim is configured to output 3D bounding box coordinates for every simulated boll in each frame. After transforming the coordinates for every boll into a shared coordinate system, the Hungarian algorithm is applied to match bolls between two frames. Matches with a large Euclidean distance (>1 cm) are excluded, as are bolls that are more than 50% occluded (as reported by Isaac). This process is repeated for every frame in the dataset, and the results are averaged to compute the overlap metric between each pair of cameras.

Using this metric, the effect of camera placement on multi-view contrastive learning performance is thoroughly investigated. To do this, all 15 combinations of two camera views were exhaustively tested. For each combination, the model was pre-trained with MoCo using either synthetic or real data from only those two cameras. The training and evaluation procedure for synthetic data were identical to that used with the real dataset, aside from some hyperparameter tweaks made to compensate for the much smaller dataset size. In particular, the MoCo momentum parameter and queue size were slightly reduced to accommodate this. In total, each model was trained for 80 epochs.

### 2.5 Downstream tasks and evaluation methods

For this study, several different downstream tasks are tested. It is found experimentally that the performance of the same self-supervised pre-training method can vary significantly depending on the downstream task, suggesting a need to test multiple such tasks. Tasks are chosen that are intended both to indicate the model's sensitivity to “global” features, as well as its ability to recognize details.

A traditional way to evaluate representation models is the linear evaluation task [30,78], where a linear classifier is trained on a supervised dataset using the generated representations as input. Since a linear classifier has such a high bias, it is likely that most of the discriminative power is coming from the generated representations. For the plot status dataset, the learned representations are evaluated directly using this method, without performing fine-tuning.

To assess the utility of the proposed method in the “real world,” a semi-supervised evaluation procedure is also employed. For these experiments, the model is first pre-trained on either the self-supervised task with real or synthetic data, COCO [79], or nothing at all. Afterwards, the YOLOv8 backbone is frozen, and the remaining layers are fine-tuned on the boll detection dataset. For these experiments, fully-supervised pre-training on the COCO dataset is used as a baseline, representing a standard transfer learning method for object detection. The “vanilla” versions of MoCo and SimCLR as proposed by He et al. [45] and Chen et al. [30], which compare two augmented versions of the same image and do not leverage multi-camera views, are also tested. These models were trained using the same datasets, data augmentation methods, and hyperparameters as were used for the multi-view versions. Because the relatively small dataset used for fine-tuning on the evaluation task can introduce variability, each evaluation training run was performed three times, and average results were reported. Overall, the variability between experiments was found to be relatively small.

A common experiment for representation learning approaches involves visualizing the latent space [80]. The goal is to verify that images that are embedded close to each-other are indeed semantically related. For this experiment, the best 3-View MoCo model is used, as it achieves acceptable results in all of the validation experiments. The model is first applied to a random image from the dataset in order to obtain its latent representation. Afterwards, the 4 other images that have representations closest to the selected image in the latent space are identified. This exercise is performed on a random subset of the pre-training data (500 images) due to computational limitations.

All experiments are performed using the YOLOv8-L model [81]. For representation learning, the model's heads are replaced with a 1x1 convolutional layer that outputs 2048 feature maps, which are used as the representation. This is a slight departure from Chen et al. [30], which used ResNet50 [82]. Similar to Chen et al. [30], however, a single-layer projection network is employed between the representation and the loss, which projects the features to a single 256-element vector using 1x1 convolution and global average pooling.

For each experiment, including the baselines, the model was pre-trained on the unannotated boll dataset for 40 epochs. The training was performed on a single Nvidia A100 GPU, using the AdamW optimizer with a learning rate of 0.001. Training hyperparameters remained constant between experiments, aside from the batch size. This was set to the largest number possible given the device's 80 GB of available memory, which varied between experiments. This necessarily means that for SimCLR, the “effective” batch size $\left(\frac{N}{V}\right)$ decreases with the number of views. For MoCo, the default queue size and momentum weight of 65536 and 0.999, respectively, were used. In general, all hyperparameters were kept at their default values whenever possible.

## 3 Results

### 3.1 Evaluation on downstream tasks

#### 3.1.1 Boll detection

**Fig. 6:**
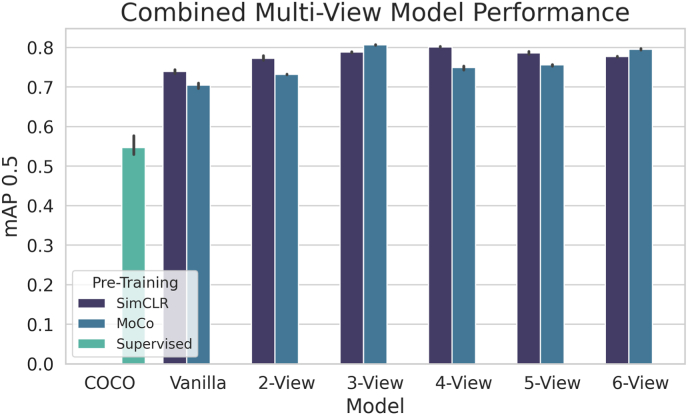
Effect of the number of views used during pre-training on the performance of the boll-detection downstream task.

**Fig. 7:**
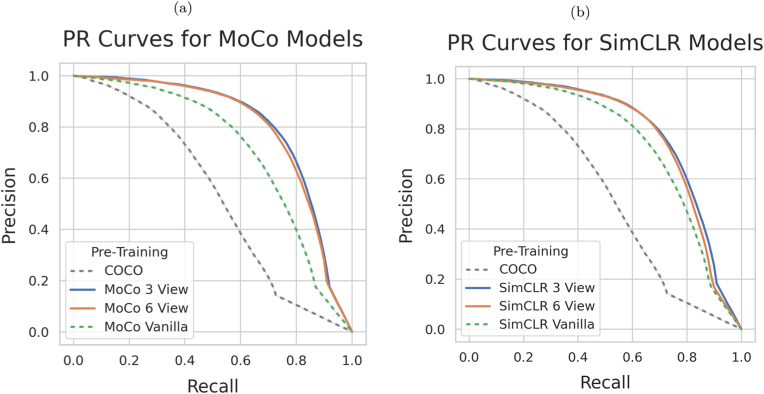
PR curves for various models with both MoCo and SimCLR pre-training, fine-tuned on the boll detection task. Dotted lines indicate baseline models, while solid lines indicate multi-view models.

In the boll detection downstream task, multi-camera views outperform both the fully supervised COCO baseline and the vanilla augmentation-based SSL methods (Fig. 6). Since there are multiple possible configurations with a given number of camera views, only the results for the best-performing configurations are reported. Performance noticeably increases with up to three views, achieving a **14%** increase over vanilla data augmentation, but then begins to saturate. This effect can be seen more clearly by looking at the Precision-Recall (PR) curves (Fig. 7). Area under the curve for the 3-view models is significantly higher than for both the baselines (dashed lines), but also marginally higher than the 6-view models. Both MoCo and SimCLR exhibit a similar trend in this regard. This suggests that the full complement of six cameras are *not* necessary to achieve good performance. With SimCLR specifically, degradation with additional views is suspected due to the decreased quality of negative samples, which arises from having to fit more camera views into the batch. With the overall batch size held constant, more cameras in the batch means fewer time steps from each camera, leading to less diverse negative samples. Configurations using both multiple cameras *and* standard data augmentation were also evaluated, but did not yield improved performance, so we only focus on multiple camera augmentation without considering standard image augmentation in this study.

Though SSL with only 3 views can match the performance of 6 views, it is important to note that this effect depends on the exact view selection. Overall, the 3-View Tight configuration (cameras 2, 3, and 5) performed the best (shown in Fig. 6), with the 3-View Oblique configuration (cameras 1, 3, and 6) performing marginally worse. Using just the three cameras on one side of the robot, however (cameras 1, 2, and 3), reduces the mAP@0.5 to **0.72**, suggesting that these camera views are too similar for effective contrastive learning. These results suggest that, so long as the spacing of views is sufficient to cover the entire plot, the *exact* spacing of the three cameras does have much of an impact on the performance of the proposed SSL approach.

#### 3.1.2 Plot status task

**Fig. 8:**
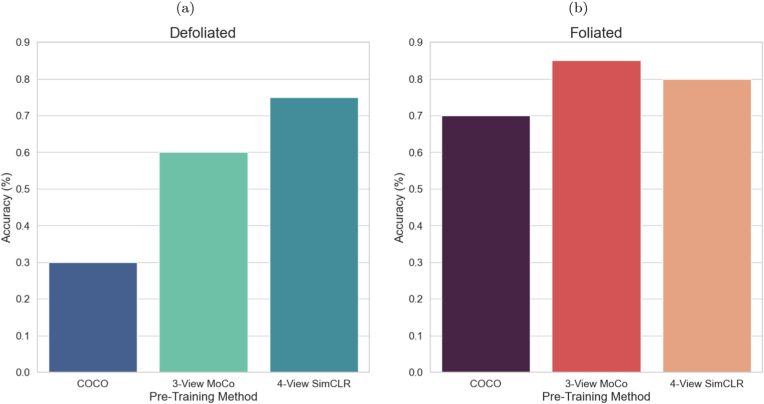
Comparison of linear evaluation results on the plot status datasets for the COCO baseline and both SSL pre-training methods. Results are shown for both the defoliated (a) and foliated (b) datasets.

**Fig. 9:**
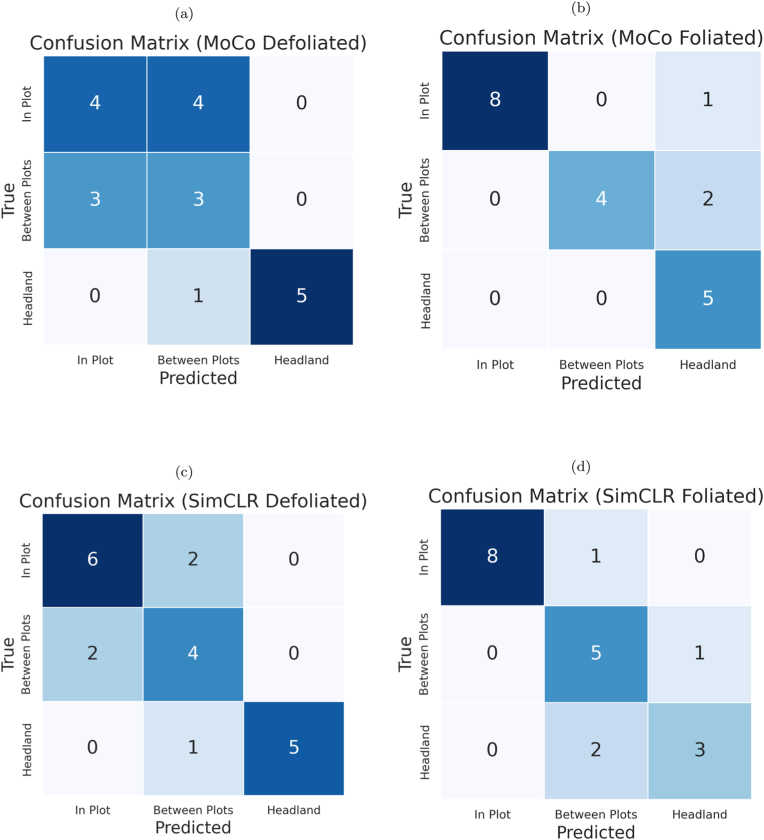
Confusion matrices for the two best-performing multi-view models on the Plot Status dataset.

When performing linear evaluation on the plot status dataset, models pre-trained through contrastive learning significantly outperform the baselines (Fig. 8). This is especially stark for the defoliated data (Fig. 8a), where the normally strong COCO baseline fails to achieve performance better than random chance. A significant domain shift exists between the COCO dataset and the cotton boll dataset, and, unlike with the foliated data, the model cannot rely on low-level color cues to identify foliage. By contrast, the best-performing models from the MoCo and SimCLR experiments perform relatively well on the defoliated data, with SimCLR slightly outperforming MoCo. Most of the mistakes made by these models appear to stem from confusion between the “in plot” and “between plots” classes (Fig. 9a and c), likely because the “headland” class is very obvious, with a complete absence of plants.

On the foliated dataset, both MoCo and SimCLR significantly outperform the baseline model with a 10% and 15% lead, respectively (Fig. 8b), showcasing the ability of the pre-trained models to generalize. Given that the model is only pre-trained on *defoliated* data, much worse performance was expected. It does help, however, that this is arguably a much easier task than the defoliated version, given that the green foliage provides a clear contrast with the ground. This is exemplified by the relatively high performance of the baseline model on this task. This also likely explains why all three models perform better on the foliated dataset than the defoliated one. In contrast to the defoliated data, MoCo slightly outperforms SimCLR here, but once again, the gap between the SSL methods and the COCO baseline is much larger than the gap between SimCLR and MoCo. These models have higher accuracy on the foliated as opposed to the defoliated data, so there is less of a clear pattern in the misclassified instances (Fig. 9b and d).

### 3.2 Effect of camera view overlap

**Fig. 10:**
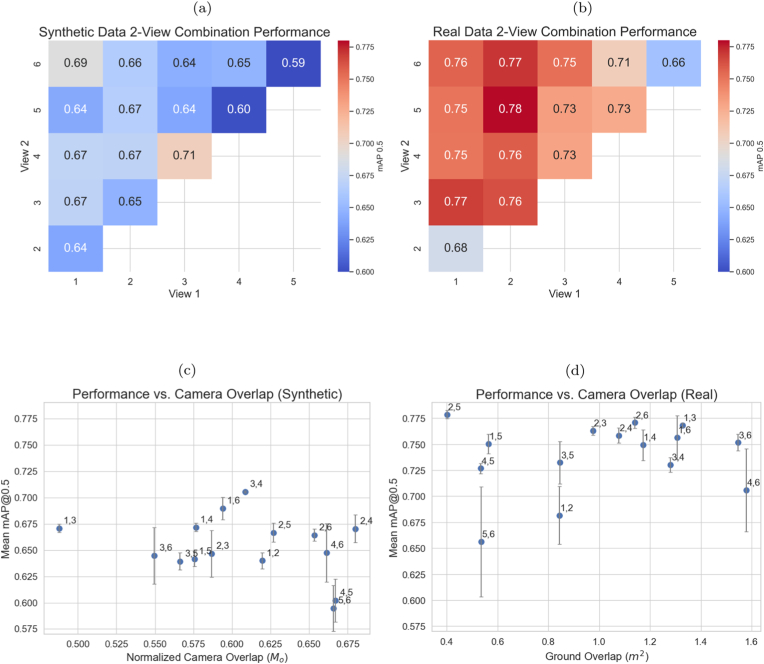
Performance of MoCo pre-training with the boll detection downstream task for every possible combination of two cameras in the synthetic (a and c) and real-world (b and d) datasets (descriptions of camera numbers can be found in Fig. 2). Performance is plotted relative to the normalized overlap metric (*M*_*o*_) for the synthetic data (c) and the calculated ground overlap area for the real data (d).

Overall, camera placement was found to have a sizable effect on contrastive learning effectiveness (Fig. 10). In general, MoCo pre-training with pairs of cameras on opposite sides (2 and 5, as well as 2 and 6, for instance) appears to be consistently effective. Additionally, some combinations of the top and side views (such as 1 and 3) perform relatively well. This holds up across both the real and synthetic data (Fig. 10), although synthetic data remains the primary focus of this analysis because it eliminates extraneous variables that might affect performance.

By contrast, some combinations perform particularly poorly. These are typically cameras that are adjacent to each-other, such as 5 and 6, or 1 and 2. It is believed that these adjacent views underperform because they carry too much mutual information [51]. One particularly interesting result, however, is that the top camera views (3 and 4), which are very similar to each-other, still manage to match the performance of vanilla MoCo on the real dataset, *even without any data augmentation*. They still perform poorly relative to the best two-view combinations (Fig. 10b), but at the very least, this seems to contradict the assertion of Chen et al. [30] that random cropping is critical for contrastive learning. The top cameras, after all, have similar fields of view. Apparently, the innate differences in the same scene captured by two different cameras are as effective as a standard data augmentation regimen [77], at least for this particular task and dataset. On the other hand, a previous study did determine that color jittering was also one of the more effective augmentation strategies (though particularly in combination with cropping) [30], and the two camera views do exhibit significant differences in color due to variations in automatic exposure and white balance. This effect is even more pronounced on the synthetic data, where the two top views are one of the best combinations. This could be because the FOVs of the two top cameras are more different in the synthetic data than the real data, as the synthetic cameras were deliberately set up to test variations in the FOV of the top views.

Overall, pre-training on synthetic data is proven to be an effective approach, despite the significant sim-to-real gap between the synthetic pre-training data and real fine-tuning data. Alignment of camera geometries between real and synthetic cameras was only approximate, mainly due to the differing geometry of the synthetic plants compared to the real ones. The synthetic cameras were configured such that a similar portion of the plants was visible as in their real counterparts, but this was necessarily inexact. Nonetheless, the contrastive learning algorithms only require there to be multiple cameras with differing views, and do not require exact alignment between real and synthetic views. Even so, there is still a noticeable gap in performance between synthetic and real pre-training (Fig. 10b and a), though it is not as pronounced as expected. In addition to the sim-to-real gap, this could also be due to the relatively small size and lack of appearance diversity in the synthetic data.

There is a relatively weak relationship between the total amount of overlap in the projected FOVs of two cameras and the contrastive learning performance of those two cameras. This measurement was performed on the synthetic data, using the normalized camera overlap (*M*_*o*_) metric defined in Eq. (2). In Fig. 10c, a weak pattern is observed, with the two best-performing camera combinations both having an *M*_*o*_ of around 0.6. However, this pattern has at least one clear outlier in the form of the high-performing but low overlap camera 1 and 3 combination. Furthermore, there are many other pairs with similar overlaps but significantly lower performance. If the amount of mutual information really is the driving force behind the performance variations among different camera combinations, then a clearer pattern in the contrastive learning performance relative to the amount of overlap between views would be expected. This more ambiguous result could be due to the trained models being too responsive to irrelevant data in the images and not responsive enough to boll visibility (in other words, the encoders are not *sufficient* and *minimal* as defined by Tian et al. [51]). This possibility is further analyzed in the remaining sections.

A similar weak pattern with outliers is also noted from the analysis of a model pre-trained on real data, with a notable cluster of high-performing camera combinations with intermediate levels of overlap (Fig. 10d). Since the *M*_*o*_ metric cannot be calculated without knowing the true location of the bolls, the camera FOVs are instead projected onto the ground based on measured camera calibration data, and the overlap between these projected shapes is used as a proxy for camera overlap. This substantially noisier measure of mutual information likely explains the weaker pattern compared to the synthetic data.

### 3.3 Exploring the latent space

**Fig. 11:**
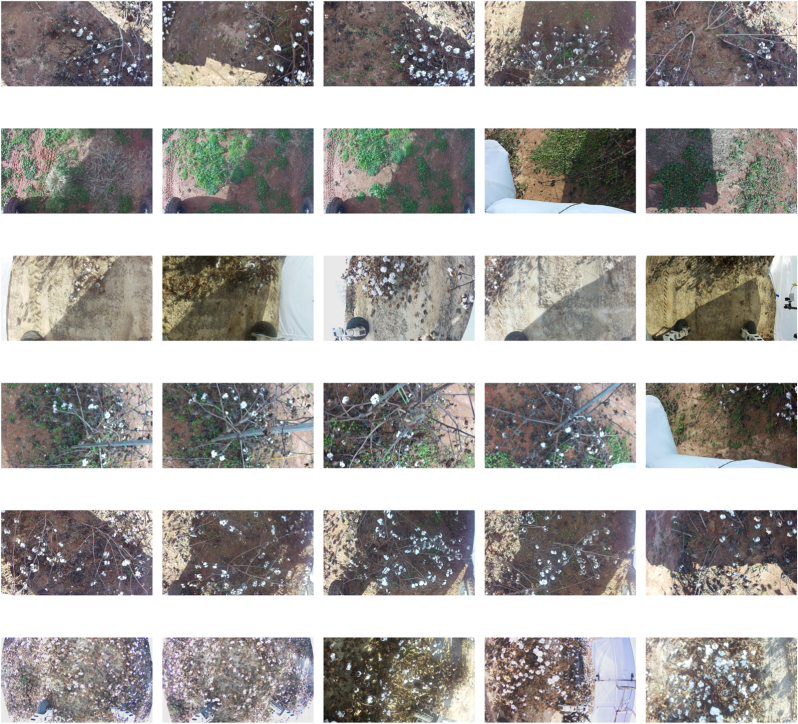
Examples of images with similar representations. The left-most column shows a randomly-sampled image from the dataset, and the other columns show the four other images that are closest to it in the latent space.

Visualizations of images embedded near each other in latent space (Fig. 11) suggest that the model effectively recognizes the “global” image structure. Generally, images that are embedded close to each other have similar lighting, and similar amounts of exposed ground, even including similar weeds. This would make sense given the model's excellent performance on the plot status downstream task, which employs similar features. In addition, the model appears to embed images of cotton in similar growth stages near each other. By contrast, it can be seen that the model is somewhat viewpoint-agnostic, sometimes embedding data from different camera angles close to each other. This is an expected and desirable property, likely stemming from the multi-view training procedure. The model's ability to pick up on details relevant to downstream tasks appears to be more limited: although it does seem to be somewhat receptive to general boll density (as can be seen in the last three rows of Fig. 11, for instance), it does not appear to be reliably clustering images based on their total number of bolls.

**Fig. 12:**
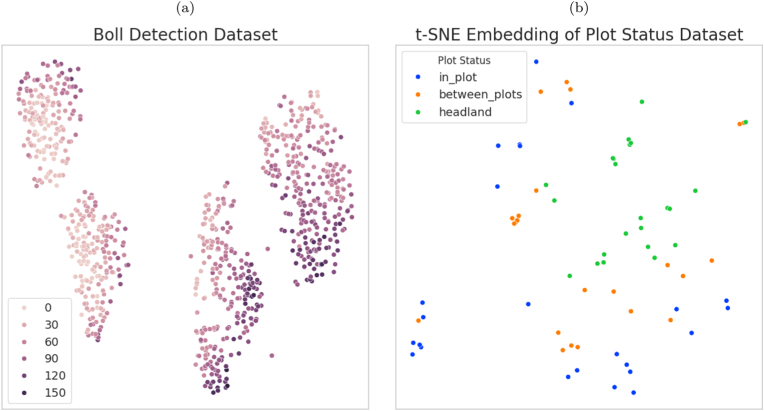
t-SNE embeddings of the representations produced by the 3-View MoCo model on the validation dataset with coloring based on boll counts (a), and on the plot status dataset with coloring based on class (b).

The semantic features affecting clustering can be inferred more easily from the t-SNE embeddings [83] (Fig. 12), which indicate that the learned representations do capture relevant features related to the downstream tasks. On the boll detection dataset within each cluster, it can be seen that the model roughly embeds images with more bolls further to the right (Fig. 12a). In the plot status data, somewhat distinct clusters for different classes are also visible, although there is overlap between the “in plot” and “between plot” classes, probably because they are fairly similar (Fig. 12b). This makes some sense, as the linear evaluation results for this task were not perfect. The two distinct clusters visible on the left and right in Fig. 12a correspond to the data from two fields that make up this dataset. This is not in and of itself problematic, as the two distinct fields do have vastly different numbers of bolls on average, so the model appears to have learned to recognize a proxy variable that correlates well with the variable of interest (number of bolls). On the other hand, the top/bottom division in each cluster appears to stem from the robot traveling in two directions as it traverses the field in a zigzag pattern. This clustering is slightly concerning, as it suggests that the model is somewhat sensitive to irrelevant criteria such as the direction of the shadows in the images.

## 4 Discussion

The alignment of pre-training dataset and downstream tasks is crucial in self-supervised pre-training. For most existing work, the standard benchmark for new self-supervised learning techniques is pre-training on ImageNet [31]. This is useful for comparing approaches to each-other and to traditional fully-supervised fine-tuning. Indeed, by this metric, recent approaches have begun to match or surpass supervised fine-tuning [28,45]. However, the ability to train on images without labels removes the need to restrict oneself to transfer learning from a few large, annotated datasets and instead allows the leveraging of internet-scale datasets. SSL has therefore become an essential approach for training generalist vision foundation models, encompassing contrastive [58,84], non-contrastive [35], and hybrid [34] approaches. On the other hand, SSL allows for the creation of specialist models as well, by pre-training from a dataset that is more relevant to the ultimate downstream tasks than the common datasets used for traditional supervised pre-training [31,79]. Indeed, the high performance of SSL on the cotton boll dataset compared to the COCO baselines provides evidence for the efficacy of this approach. Nonetheless, comparison in isolation to COCO representations is insufficient to ascertain the performance and annotation trade-offs of the proposed method relative to fully [12,[85], [86], [87]] and semi-supervised [2] alternatives. Future work should focus on making systematic comparisons with such approaches.

The specific downstream tasks were chosen as representative benchmarks for precision agriculture. Cotton boll detectors, for instance, are a critical part of high-throughput yield estimation pipelines [[86], [87], [88], [89], [90]]. Similarly, the plot status task is a simplified version of visual navigation tasks for field robots. The primary goal of this SSL approach is to make it feasible to construct such models with minimal manual data annotation, which tends to be the primary labor bottleneck with deep learning. This study presents a reasonable proof-of-concept for that idea. For instance, the boll detector used in this study was trained on only 116 images, and achieved acceptable performance. By contrast, Tan et al. [88], operating on similar data but training without SSL, used a total of 693 training images. This reduction in annotation burden provides a tangible benefit to researchers.

The proposed method differs from previous work [30] in that it uses the YOLOv8 backbone to extract representations instead of ResNet [82]. This design choice was motivated mainly by the boll detection downstream task. Previous work [30,45] primarily uses ImageNet [31] classification as a downstream task, which ResNet is well-suited for. However, YOLO models are generally a better choice for object detection, and are widely-used for actual cotton yield estimation pipelines [72,89,91]. As such, the YOLO model was chosen in this study in order to mimic an engineering workflow that could conceivably be used to train a boll detector in the process of deploying an HTP system in the field. It is believed that this represents a more realistic use-case for the chosen application domain.

Synthetic datasets are also a potentially viable option for SSL pre-training, but only in situations where annotations are not available. Even though this was not the goal of this study, the better-than-expected results from pre-training with synthetic data and fine-tuning on real data somewhat mirror supervised transfer learning experiments, in which a mostly synthetic dataset with a small amount of real data can achieve acceptable performance on real data [92]. In reality, many synthetic data generation frameworks support generating annotations directly; in this case, a more traditional fully-supervised transfer learning pipeline probably makes more sense. However, the ease of generating annotated data mostly applies to traditional rendering-based approaches. More advanced techniques such as GANs [64] and diffusion models [65] might be able generate much more realistic synthetic data that nonetheless lacks annotations. Current approaches for leveraging such data can be sub-optimal [62]. In such cases, SSL pre-training could be useful. For this study specifically, it should be noted that, due to the gap between synthetic and real data, the primary purpose of the synthetic data analysis is exploratory. That said, these results suggest a relationship between camera overlap and SSL performance, which might warrant additional exploration with real camera data.

Contrastive learning from multi-camera views is an important aspect of this work that has previously been under-explored. Tian et al. [48] provide a framework for contrastive learning with more than two views, and show that increasing the number of views can be beneficial. The results of this study align with this older conclusion, to a point. More views are beneficial as long as they contain the proper amount of mutual information [51], as evidenced by the performance saturation that was observed with a large number of similar views. The proposed approach also has the advantage of being input-agnostic: it could potentially be applied to other crops or other organs of the same crop (such as flowers instead of bolls). Technically, the dataset does not even have to be uniform in the number of cameras, as the proposed loss allows for variations in the number of views between batches, or even supplementing examples that have fewer views using augmentations of existing ones.

Though the results are promising on the cotton boll dataset, additional research is needed to determine the efficacy of this approach on other crops. Unfortunately, the majority of existing public datasets do not contain synchronized imagery from multiple cameras, which is a requirement for the proposed approach. Given that there are already existing ground-based phenotyping platforms with multiple cameras [73,86,93], the lack of multi-camera datasets is likely not due to a paucity of relevant data being collected, but more due to the data not being released publicly. By demonstrating the utility of these data, even in unannotated form, perhaps this study will inspire future researchers to release more such datasets. In the meantime, future work will focus on collecting more data using the MARS-X platform with other crops. The use of multiple cameras (and the possible addition of auxiliary modalities such as near-infrared or depth) should provide large, high-quality datasets for testing the generalizability of the proposed approach.

Future research is needed with regards to the optimal camera configuration for multi-view SSL. Though the clear differences between different combinations of views are intriguing, the ultimate goal would be a method of predicting the effectiveness of two arbitrary views in a contrastive learning setup based on their camera parameters. Though the current work can provide some guidelines on designing multi-camera systems for contrastive learning, the ultimate results of the analysis from this study are less clear-cut: the camera overlap metric that was introduced does not correlate strongly to performance. The results therefore suggest only a tentative relationship between view overlap and downstream task performance which may be influenced by extraneous factors. Specifically, different crops, downstream tasks, and imaging platforms could yield significantly different results in this analysis. More investigation in this vein could potentially provide more clarity. Additionally, by the standards of modern foundation models, the smaller-scale experiments in this study look to be merely a starting point. It is quite possible, even likely, that scaling up the model and dataset size will yield significant improvements [94].

More advanced SSL approaches could also be tested with the proposed multi-view learning paradigm, replacing or augmenting SimCLR and MoCo. In particular, non-contrastive approaches such as Masked Auto-Encoders [28] are competing with contrastive learning as the dominant paradigm for image-based SSL. It seems, however, that combining contrastive and non-contrastive approaches can surpass either method alone [34,57], an effect suggested by theoretical analysis of multi-view SSL [95]. As such, a hybrid, multi-task approach, which maintains the contrastive loss but also adds a non-contrastive reconstruction loss, seems like a particularly promising direction for future research.

Though the proposed self-supervised approach learns useful, relevant features, some concerning evidence suggests that it also learns irrelevant features related to lighting and field configuration. Ultimately, this likely arises from the nature of the datasets, which typically exhibit a multimodal distribution comprising data from different collection sessions. An SSL approach might learn to group examples by session instead of by features that really matter. Guiding contrastive learning techniques towards learning useful representations and not irrelevant ones is an unsolved problem, but it is typically believed that the selection of negative and positive pairs has a large effect [30,51]. Unfortunately, these types of biases are likely inherent to the contrastive learning objective, seeing as it merely guides the model towards separating the dataset into groups without enforcing that the grouping be semantically meaningful. Non-contrastive approaches such as Masked Autoencoders [28] may avoid this particular issue but undoubtedly suffer from their own representational biases.

Previous contrastive approaches [30] have relied on aggressive data augmentation to avoid learning poor representations, but this likely works better with datasets such as ImageNet which exhibit a more uniform distribution. By contrast, it was found that data augmentation *in addition to* multi-camera view selection yielded little benefit for the proposed approach. Nonetheless, only SimCLR-style augmentation was explored; perhaps more advanced techniques such as CutMix [96] and MixUp [97] could help by combining multiple images from the dataset into a single example, thus flattening the troublesome multimodal distribution somewhat. Alternatively, perhaps the selection of negative examples could be explicitly biased towards images from the same “spurious” group by periodically evaluating the model's representational biases during training and adjusting the selection algorithm accordingly. This idea is conceptually similar to ContrastiveCrop [50]. Furthermore, the notion that modern Mixture-of-Experts techniques [98,99] might neutralize this issue by learning to specialize on different, related sub-groups of the datasets also warrants further investigation.

## 5 Conclusions

Overall, multi-camera self-supervised learning holds promise as a technique for reducing data requirements within the context of automated plant phenotyping. A large, unannotated dataset related to the target task can be leveraged for this purpose. The effectiveness of this approach is, however, significantly affected by the exact choice of camera views, with some evidence to support the idea that intermediate amounts of overlap are effective. Though the dataset included six cameras, it was found that, generally, fewer cameras, properly placed, can achieve similar performance. SimCLR and MoCo both perform similarly, with some small discrepancies for specific tasks. Through analysis of the representations, evidence was found that the model learns to capture semantically-meaningful features, but may be affected by some inconsequential image variations. It is hoped that in the future, by scaling up the model and dataset size, it will be possible to further increase performance. Furthermore, the effectiveness of the approach on other crops will also be investigated.

## Author contributions

• **Daniel Petti:** Conceptualization, Data Curation, Formal Analysis, Investigation, Methodology, Software, Visualization, Writing (Original Draft)
• **Changying Li:** Conceptualization, Funding Acquisition, Project Administration, Resources, Supervision, Writing (Review and Editing)
• **Ninghao Liu:** Methodology, Supervision, Writing (Review and Editing)

## Declaration of competing interest

The authors declare that they have no known competing financial interests or personal relationships that could have appeared to influence the work reported in this paper.

## Data Availability

The processed data required to reproduce the above findings are available to download from 10.5281/zenodo.18164649. The code required to reproduce the above findings are available to download from https://github.com/UGA-BSAIL/self-supervised-learning.
